# Analyzing protest mobilization on Telegram: The case of 2019 Anti-Extradition Bill movement in Hong Kong

**DOI:** 10.1371/journal.pone.0256675

**Published:** 2021-10-08

**Authors:** Aleksandra Urman, Justin Chun-ting Ho, Stefan Katz

**Affiliations:** 1 Social Computing Group, University of Zurich, Zurich, Switzerland; 2 Institute of Communication and Media Studies, University of Bern, Bern, Switzerland; 3 School of Social and Political Science, University of Edinburgh, Edinburgh, United Kingdom; 4 Centre for European Studies and Comparative Politics, Sciences Po, Paris, France; 5 Polyflow GmbH, Zurich, Switzerland; University of Albany, State University of New York, UNITED STATES

## Abstract

Online messaging app Telegram has increased in popularity in recent years surpassing Twitter and Snapchat by the number of active monthly users in late 2020. The messenger has also been crucial to protest movements in several countries in 2019-2020, including Belarus, Russia and Hong Kong. Yet, to date only few studies examined online activities on Telegram and none have analyzed the platform with regard to the protest mobilization. In the present study, we address the existing gap by examining Telegram-based activities related to the 2019 protests in Hong Kong. With this paper we aim to provide an example of methodological tools that can be used to study protest mobilization and coordination on Telegram. We also contribute to the research on computational text analysis in Cantonese—one of the low-resource Asian languages,—as well as to the scholarship on Hong Kong protests and research on social media-based protest mobilization in general. For that, we rely on the data collected through Telegram’s API and a combination of network analysis and computational text analysis. We find that the Telegram-based network was cohesive ensuring efficient spread of protest-related information. Content spread through Telegram predominantly concerned discussions of future actions and protest-related on-site information (i.e., police presence in certain areas). We find that the Telegram network was dominated by different actors each month of the observation suggesting the absence of one single leader. Further, traditional protest leaders—those prominent during the 2014 Umbrella Movement,—such as media and civic organisations were less prominent in the network than local communities. Finally, we observe a cooldown in the level of Telegram activity after the enactment of the harsh National Security Law in July 2020. Further investigation is necessary to assess the persistence of this effect in a long-term perspective.

## Introduction

In the beginning of 2021 Telegram, a privacy-focused messaging app, has reached 500 million active users, becoming more popular than Reddit, Twitter, Quora and Snapchat [1] as well as the most downloaded app worldwide in January 2021 [2]. The messenger is becoming increasingly popular across the globe. Nonetheless, there is little research in relation to this platform to date, and the existing few studies deal primarily with the dissemination of extremist content on the app [3–5]. Telegram, however, is not only used by political fringe groups—though its privacy focus makes the messenger especially attractive to them [3]. This same privacy orientation, in part explained by the background of Telegram’s founders, is what also explains the messenger’s popularity with users in authoritarian states such as Iran or Russia.

The messenger was created by two entrepreneurs of Russian origin—Pavel and Nikolai Durov—after they fled Russia due to legal issues with the Russian state over freedom of speech. Years before launching Telegram, in 2006, the Durov brothers had founded Vkontakte (vk.com; aka “Russian Facebook”) which soon became the most popular social media platform in Russia and other post-Soviet countries. Durov has always claimed to be a keen advocate of user privacy. Being the CEO of Vkontakte, he repeatedly refused to take down Vkontakte pages of Russian oppositional activists and cooperate with the Russian authorities in other ways (e.g. provide them with access to users’ data). In early 2013, months before Telegram was launched, Durov’s place was raided by the police. After that the entrepreneur spent months in hiding. In June 2013, Edward Snowden came forward with his revelations about the NSA, which, according to Durov, strongly affected him: “We learned that [security] isn’t just our problem in Russia, it is a global concern.” [6]. In January 2014 Kremlin-linked investors took control over Vkontakte, and the Durov brothers sold their shares and had to flee Russia. Since then Durov has been living in exile [7] and focusing on the development of Telegram.

Telegram, thus, was founded by privacy-keen entrepreneurs amid their own troubles with the authoritarian Russian state over the freedom of speech and Snowden’s revelations. It comes as no surprise that the platform is privacy-focused which is underscored in its FAQ: “…if criticizing the government is illegal in some country, Telegram won’t be a part of such politically motivated censorship. This goes against our founders’ principles. While we do block terrorist (e.g. ISIS-related) bots and channels, we will not block anybody who peacefully expresses alternative opinions” [8].

Telegram has also demonstrated sturdiness in light of attempts to block it in authoritarian states. One such unsuccessful attempt took place in Russia in 2018 after Telegram refused to give Russian authorities access to user data. In response, Durov called for “digital resistance”—an initiative that, according to Telegram’s spokesman, is “a community of anonymous developers who created proxy servers around the world to help Telegram remain accessible to Russian users” [9].

Durov and Telegram openly support protests in authoritarian states such as, most recently, the protests in Belarus [10]. Durov’s commitment to users’ privacy, Telegram’s sturdiness in the face of the attempts to block it and Durov’s support for the protests have undoubtedly contributed to the app’s credibility in the eyes of the protesters in authoritarian countries and, possibly, beyond.

From a technical point of view, crucially for political activists in authoritarian regimes, Telegram combines “enhanced privacy and anonymity along with the opportunities to gain publicity (through channels) and coordinate and mobilize (through groups)” [3]. Most of the app’s features—i.e., public and private channels, public and private groups, encryption, secret chats, unsend anything and anonymous forwarding—have been outlined by Urman and Katz [3] in detail, along with the reasons why these features are helpful to users who want to gain publicity while preserving anonymity.

In addition to the functions mentioned in [3], other features of Telegram that are of high importance in the context of protests are the absence of algorithmic filtering, advertisements and the difficulty of searching for new channels on Telegram. Due to the absence of algorithmic filtering, users are in control of what they see on the app. Since channels and groups are difficult to search for, their growth and popularity depend on them being referenced by other—already popular—Telegram sources. Along with the absence of paid-for content promotion, this means that it is more difficult for authoritarian states to spam and hijack conversations on Telegram compared to other platforms such as Facebook or Twitter. Finally, as the example of Russia—and, more recently, Belarus—shows, Telegram is difficult to block—a feature highly relevant for protesters who fight against repression by authoritarian governments.

Despite Telegram’s popularity with protesters around the world, there are no studies that would examine protest mobilization on the app. We find this is a major research gap. The platform’s affordances are very different from those of Facebook or Twitter and Telegram-based protest mobilization might function very differently from the one that took place on the other platforms. For instance, personalized public sharing—the foundation of so-called “connective action” [11]—does not play as much of a role on Telegram due to the way the platform is organized. One sees either personal messages from their friends or posts from public channels one has subscribed to in their feed, not what their friends have publicly reposted—the latter feature is absent on Telegram.

In this paper, we scrutinize the way protest mobilization takes place on the platform using the Hong Kong protests of 2019—one of the largest Telegram-aided pro-democratic movements—as a case study. With this paper we aim to provide an example of methodological tools that can be used to collect and analyze Telegram data to study protest mobilization and coordination on the platform. Methodologically, we also contribute to the research on computational text analysis in low-resource Asian languages—in this case, Cantonese. Finally, this study adds to the body of existing literature on the 2019 protests in Hong Kong.

## Case study: 2019 protests in Hong Kong

In 2019, Hong Kong experienced massive Anti-Extradition Law Amendment Bill (Anti-ELAB) protests. They were first triggered in the end of March 2019 by a proposed bill that would have allowed Hong Kong to send fugitives to jurisdictions without existing bilateral extradition agreements with Hong Kong, including Mainland China, Taiwan and Macau. The proposed bill was said to be prompted by a murder case in Taiwan in 2018. Still, the fact that the proposed legislation, once passed, would allow extradition to China has raised considerable doubts about the government’s motivations. Despite the widespread public concerns, the bill was set to pass the second reading in the Hong Kong’s Legislative Council (LegCo) on June 12. In response, tens of thousands of protesters surrounded the LegCo and forced the cancellation of the meeting. Although the government announced the suspension of the bill on June 15, the protest evolved to incorporate other demands including the complete withdrawal of the bill and the retraction of the riot characterization of the June 12 protest [12]. The protests were marked by police brutality, attacks on protesters by pro-government groups, and eventually turned into a series of recurring weekly protests until early 2020 when the COVID-19 pandemic brought a recess [12]. At times protests would turn violent resulting in damaged infrastructure and physical clashes between protesters and the police during which both sides as well as bystanders suffered injuries, some of which were grave [13]. After the start of the pandemic, the political climate has become even more unfavourable to the protests after China imposed a harsh Security Law on Hong Kong on June 30, 2020, under which secession and subversion activities are punishable with up to life imprisonment.

The Anti-ELAB protests have often been described as leaderless and decentralized [14–16]. In Hong Kong, there has been widespread antipathy among protesters towards a centralized movement leadership since the later stage of Umbrella Movement in 2014. As a result, the idea of “no central stage” (無大台) has been foundational to the 2019 Anti-ELAB protests since its early stage [12]. In the absence of a leading organisation, protesters coordinated their activities through encrypted messaging apps, most notably Telegram, as well as online discussion forums [12, 17, 18]. These platforms allowed a high volume of information flow and strategic discussions to take place while the participants remained anonymous [12]. There are no precise numbers on how many people in Hong Kong actively used Telegram during the protests of 2019, however the data from a private company SensorTower shows that the downloads of the Telegram’s app on mobile soared in the summer of 2019 when the protests erupted [19]. The surge in the number of installations of the app was similar to the increase in the downloads of the app LIHKG—a Reddit-like discussion forum that has been demonstrated to have been crucial to the Anti-ELAB protest mobilization online [18, 20]. Yet Telegram remains an uncharted territory despite its importance in the protests both in Hong Kong in 2019 and beyond—i.e., Belarus in 2020 or Russia in 2019.

## Research questions

The main goal of the present study is an explorative analysis of the Telegram-based activity related to the 2019 protests in Hong Kong. Telegram was used by the protesters to coordinate their activities [21] but the dynamics of the use of Telegram during the protests is unclear. For instance, at what point Telegram, previously not very popular in Hong Kong, became the main platform for the protesters, and how fast the movement grew on the platform.

*RQ1: What were the dynamics of Hong Kong protest movement’s development on Telegram?*

One of the most important mechanisms through which social media can positively affect protest mobilization is the facilitation of the spread of protest-related information, motivational and coordinational messages [22, 23]. However, the efficient spread of information is crucial for this mechanism to have influence. In the context of social media, potential efficiency of information spread can be assessed with the help of network theory [24]. In practice, online networks are often sparse and fragmented. Such structures hamper the diffusion of information in a network, thus decreasing the potential of social media-based mobilization [24]. For it to be maximized, there should be actors that act as structural bridges who facilitate information diffusion across different communities in the network. Ideally, such information diffusion across communities would lead to increased cohesiveness—that is, less fragmentation,—of the network making information spread more effective.

The basis for information diffusion on Telegram is formed by public channels and groups that are not easily searchable due to the way the platform works and whose discoverability and, thus, reach directly depends on them being cited by other channels. Hence, in the case of Telegram, the analysis of the coherence of a citation network related to a certain topic or movement is particularly important for the assessment of the efficiency of information diffusion. We address this with the second research question:

*RQ2: How fragmented was the citation network of public Telegram channels and groups related to the Hong Kong protest movement of 2019?*

Not only the efficiency of information diffusion is essential but also the content that is being spread. For protest movements, information related to police presence, availability of medical and legal help, and coordination-related messages is especially relevant [22]. In addition, emotional messages related to the protests are helpful for mobilization [22]. For this reason, we also examine the content of the messages spread on Telegram and pose the third research question:

*RQ3: Which topics were discussed by political activists on Telegram in relation to the Hong Kong’s protests of 2019?*

Political communication scholars have claimed that social media create opportunities for the development of “leaderless” protest movements [11, 25]. Yet, empirical research on social media-enabled protests of the early 2010s shows that leaders remain important for such movements both online, and offline [26, 27] even if contemporary protesters prefer portraying the movements as “leaderless” [27].

On Telegram, in the absence of a public feed sharing function, the major means for coordination and communication are public channels and groups. Groups are essentially group chats where anyone can post, while channels are normally administered by few people, and only these people—channel administrators—can post to a given channel. This structure is strikingly different from that of Facebook and Twitter and, at the first glance, is perhaps more conducive to the emergence of leaders (i.e., specific channels coordinating protest actions) rather than to the development of a leaderless protest as the 2019 protests in Hong Kong were according to the previous research [21]. We aim to scrutinize the claim about the leaderlessness of the movement with the following research question:

*RQ4: What are the dynamics of leadership in Telegram-based activities during the 2019 Hong Kong protests?*

Telegram has proven to be somewhat immune to the Internet-based measures set to curb its influence, as unsuccessful attempts to block it in Russia and Belarus show. However, online censorship is not the only possible way of curbing Internet-based protest mobilization. On June 30, 2020, China imposed a harsh Security Law on Hong Kong, under which secession and subversion activities are now punishable with up to life imprisonment [28]. The law has created a chilling effect across Hong Kong: pro-democracy groups, including Demosisto founded by one of the most famous pro-democracy activists Joshua Wong, were disbanded; several prominent pro-democracy activists, including Nathan Law, fled Hong Kong; Hong Kong people took down their Twitter and Facebook accounts; shops removed “Lennon Walls”—walls made up of Post-it Notes bearing pro-democracy messages to show support of the protests; and journalists have erased their names from digital archives [29–32]. Hong Kong Telegram users, particularly channel administrators, have become the targets of multiple arrests in the past [33–35], which has prompted the app to push changes to protect the identity of Hong Kong protesters [10]. Against this background, even though Telegram was not targeted specifically by the Security Law, it is possible that the enactment of the Law has affected Telegram-based activities as well. If that is the case, the example of Hong Kong would set a troubling precedent in the context of social media-enabled mobilization in autocratic regimes, suggesting that even privacy-focused platforms that successfully resist online censorship might not be an effective tool in a fight against a powerful authoritarian regime if it takes certain measures aimed at curbing mobilization in offline as well as online sphere. In connection to this, we examine the final research question of the present study:

*RQ5: Did the enactment of the Security Law affect the activities of Hong Kong’s political Telegramsphere?*

In the following sections, we describe the data and methodology used in the analysis, present the results and discuss their implications.

## Data and methods

### Data collection

We collected the data from 1806 public Telegram channels from the Hong Kong Telegram ecosystem. The data was collected using Telegram’s open API and Telethon Python library [36] using Exponential Discriminative Snowball sampling [37–39]. The data collection is in compliance with the Telegram’s terms and conditions. The Human Subjects Committee of the Faculty of Economics, Business Administration and Information Technology at the University of Zurich has confirmed that the study would have been exempt from the ethics approval review under the current regulations. No private or personal identifiable data was used in the analysis.

Our methodology is similar to that described in a study on far-right Telegram channels [3]. As Telegram’s API is not searchable—i.e., one can not search for specific keywords or tags,—snowballing-based techniques are the only way to sample a large amount of data on a specific topic without manually predefining the list of channels to collect the data from. Such manual sampling might be appropriate for some studies, especially when the authors are interested in specific actors. However, in the case of the present study that aimed to have a rather comprehensive sample of channels and public groups dedicated to the Hong Kong protests such manual sampling was not feasible. There are no curated lists of channels and groups related to the Hong Kong protests that we could build on to create a list for crawling. Further, as Telegram is not easily searchable, the creation of such a list by the authors would have inevitably led to the omission of some channels and/or public groups. Thus, we relied on a snowballing-based technique, and believe that for future studies on Telegram similar methodologies will be useful in the absence of curated lists of groups and channels. We collected only public data (i.e., no private groups/channels/messages data was collected; the only information available to us was that set as public by the channels owners or public group creators (i.e., channel/public group names that do not allow identifying who the channel creator/administrator is), or by the users (i.e., their usernames only if set to public; these, however, can be changed by the users at any point in time and do not allow identifying individual users). Thus, no private or personally identifiable information was collected or used in the analysis. If a channel or a group changed a username, we treated the old and the new usernames as two distinct channels: it is plausible to assume that the change of a username at least temporarily decreases a channel’s recognition. Further, our tests running different parts of the analysis showed that the conclusions are not affected regardless of whether we treat channels that changed usernames as distinct vs identical. Hence, we opted for relying on usernames as, in the case of non-sourced channels and groups (those that were mentioned but whose messages were not collected) it was not possible to infer their persistent IDs rather than usernames, and for individual users who were mentioned we did not want to rely on IDs for privacy reasons to minimize the data collected about individuals: while usernames can be changed and are only visible if a user created a username and set it to public, IDs are persistent and exist for all users.

The fact that we collected only public data, though, is an important limitation within the context of the present study: it is unclear whether discussions in private group chats unobserved by us were of fundamentally different nature than those in the public ones. Though there is no way to answer this question using our methods, it is necessary to keep in mind when interpreting our results, and, we suggest, this question would be worthwhile to explore in the future using different methodology such as conducting in-depth interviews with Telegram users who were active in both, private and public chats, during protests in Hong Kong or other countries such as Belarus and Russia. Another important limitation related to the usage of public Telegram groups is that those could have been infiltrated by the law enforcement [40]. We suggest though that this should not have affected the results of the aggregate analysis in a major way as the alleged infiltrators reportedly used the chats to spy on the protesters activities, not to post own messages [40], and even if the latter was the case, the volume of their messages would have to be very large to affect our results which is unlikely.

Unlike in traditional sampling, the initial seed was not sampled randomly but chosen by us—a method appropriate to reach hidden populations with specific properties [41]. As the initial seed we chose the channel @dadfindboy, which at the time of the protests was one of the most prominent channels among Hong Kong activists according to tgstat.com. From that channel, we relied on snowballing as follows: selected the most mentioned channel in the seed’s messages, and collected all of its messages; then, we collected the data from the channel most mentioned by the seed, and the channel collected after it; this procedure was repeated many times. In total, we collected full histories of 1806 public channels and groups. By “full” histories we mean all the messages that were available at the point of data collection. Since one can “unsend” messages on Telegram, some of the messages could have been deleted—similarly to how public posts can be deleted on other social media such as Twitter or Facebook. We, however, suggest that in principle even if some messages were deleted, our conclusions would be unaffected by this as our analysis is aimed at providing an overview of the main trends on the aggregate level. Based on the collected full histories, we then constructed a directed citation network of Telegram sources. In this network, a node corresponds to a Telegram channel or public group chat. An edge between two nodes corresponds to a mention/repost with the weight being equal to the number of mentions/reposts (i.e., if channel A mentions channel B 3 times during the observation period, there is a directed edge from A to B with weight 3). In total, the resulting full network includes 1806 nodes with full histories (“source nodes”), 58,563 nodes mentioned by them for which full histories were not collected, and 353,386 weighted edges.

### Methods

To answer RQ1 and assess how the observed citation network developed, we first examined the rates of its growth. As proxies to measure this, we used the number of edges, the total number of source nodes and the number of newly added source nodes in the network observed per month. New source nodes here correspond to the channels that became active within a specific month. For this analysis, we used the observation period starting in January 2019—three months ahead of the introduction of the Extradition Bill that triggered the start of the protests in Hong Kong in the end of March 2019.

To address RQ2, we looked at the level of network fragmentation in different months. For this, we computed modularity scores using Louvain algorithm [42] for the snapshots of the network, each snapshot corresponding to one month of observation. Given the duration of the protests, we deemed snapshotting by month the most appropriate. However, if one examines different Telegram-based movements using similar methodology, snapshotting by shorter periods might be recommended—i.e., by week, day or even hour, depending on the exact event under examination.

To answer RQ3 and evaluate the content that was shared on Telegram and discussed by the protesters, we employed quantitative text analysis techniques. Due to the high number of messages posted on Telegram, it would have been difficult in terms of the necessary computational resources to run analysis on the full sample. For this reason, we decided to restrict the analysis to the most important channels in the observed citation network. For that, we have applied the HITS algorithm [43] to the network and identified the twenty nodes (channels) with the highest Hub and Authority scores. Channels with high authority scores serve as important sources of the primary content and are often cited by the others; those with high hub scores, on the other hand, function more like directories of useful information from other sources [43]. Hubs heavily link to authorities, but are not necessarily often linked to; authorities are frequently cited by hubs, but do not necessarily cite other channels. By including both types in our analysis, we were able to encompass both types of sources—authorities who might have acted as coordinators or main sources of new information and hubs that might have served as aggregators of information from different sources as both types are important in the context of protest mobilization.

A total of 3,558,662 messages were extracted. Computational text analysis in Cantonese is a non-trivial task due to the absence of white space in Cantonese syntax. Researchers therefore have to rely on tokenisers to split texts into basic word units to allow for further analysis [44]. However, the majority of currently available resources were developed for Mandarin, another dialect of the Chinese language, and therefore perform poorly on Cantonese texts. We hope that the methodology description below can serve as a useful reference for future studies that use automated text analysis techniques in application to the texts in Cantonese.

**Fig 1:**
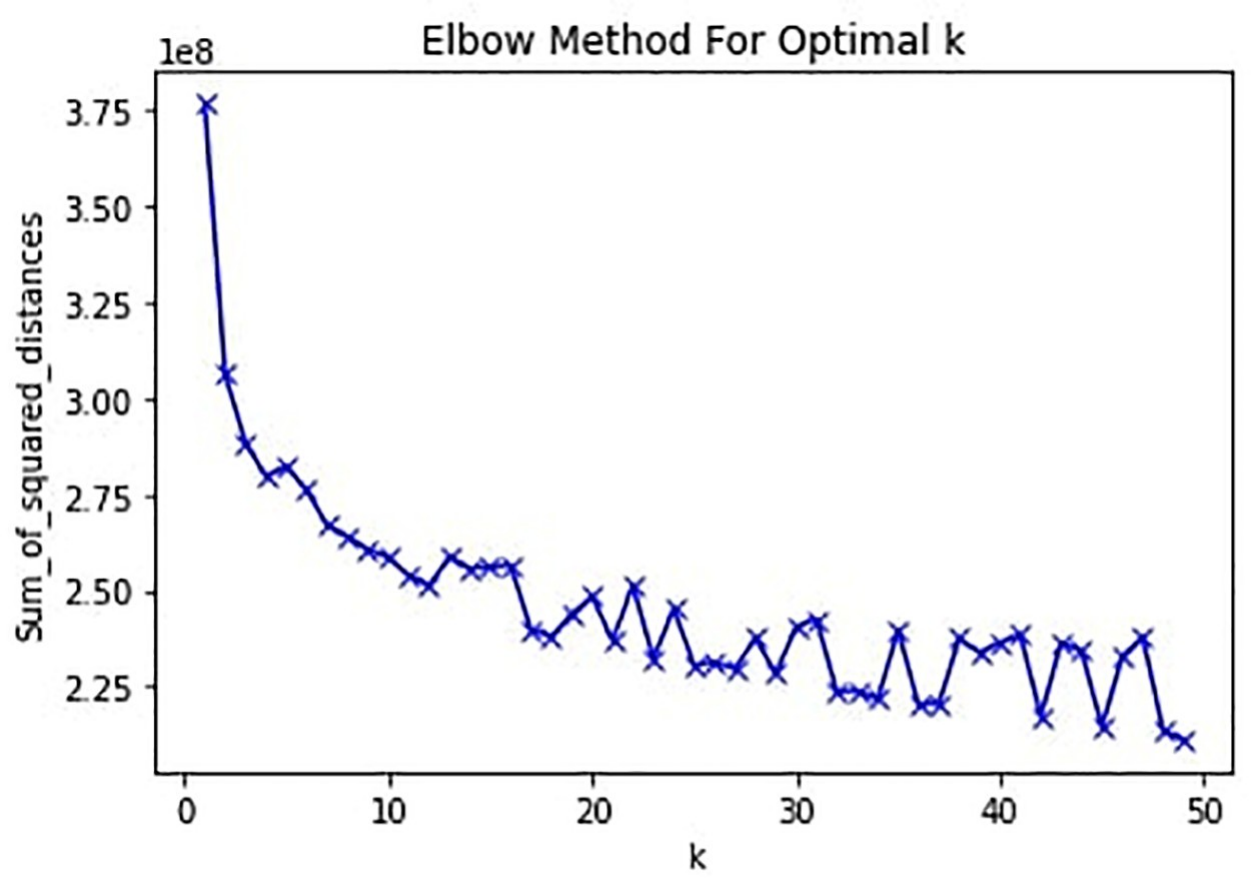
Elbow method for optimal k.

To proceed with tokenisation, we employed a method that is commonly adopted for finding multi-word expressions in other languages. We used the phrase (collocation) detector implemented in the Gensim python package to detect the characters that appear frequently together but infrequently when separated [45, 46]. We then extracted the word list and read manually to determine if they are valid Cantonese words. Eventually, the list was then used to supplement Jieba Chinese tokeniser [47]. The word list is available at [48]. We then used the tokenised words to train a continuous bag-of-words (CBOW) model using Word2vec. CBOW is a neural network-based algorithm to learn the underlying vector representations of words in the high-dimensional vector space [49]. In the model, each word is represented by a 100-dimensional vector where words that have similar meaning will have similar representations in the vector space. Document embeddings were then calculated by averaging all the words within each message. To examine the topics within the corpus and their prevalence, we used an unsupervised k-means clustering method to cluster the document embeddings [50]. The optimal number of k was determined through the within-cluster sum of squares elbow method for 1 to 50 clusters (Fig 1). Following this, we used a k-means method with *k* = 12, where each cluster represents a topic within the contents. We then validate the clustering by manually reading random samples of messages from each cluster until saturation is reached.

To answer RQ4, and establish whether there is evidence of the presence of specific leaders, we have repeated the HITS-based analysis for 8 monthly snapshots (from May 2019 to December 2019), in each case taking into account only the messages posted within a specific month, and constructing a monthly snapshot of a citation network. For each monthly network, we extracted top-10 channels by Authority and Hub scores, and then compared their position in terms of Authority and Hub scores to other months. We suggest that the dominance of a single or few nodes throughout the duration of the protests would indicate that those nodes acted as de-facto leaders of the protest. The absence of such dominance would indicate that there were no specific organized leaders, with different groups taking over at different times and suggesting that the protest was in fact leaderless—at least in the traditional sense of leadership characteristic for collective movements of the past in the context of protest coordination and information spread-induced mobilization within Telegram. One limitation is that this method does not account for the potential leadership role of specific individual users. For example, if a user or several users administered different Telegram channels which, in turn, were switching in the observed importance, they would be indeed protest leaders. However, as the data on channel administrators is not public, it is not possible to assess the role of individual users on Telegram. We thus can only assess the role of organized groups or channels, not individuals. This is a limitation that can not be overcome due to the nature of the data but should be accounted for when interpreting the findings.

To check whether the National Security Law introduced in July 2020 has significantly affected the activities within the observed Telegram network and thus answer RQ5, we employed Interrupted Time Series Analysis [51] applied to the daily rates of edge formation in the observed network. We examined the period from March 2020 to the end of September 2020. The cut-off point for the start of the observation was chosen to isolate the effects of other major events such as the beginning of the COVID-19 pandemic or the protests in Hong Kong in 2019 on the timeseries. In total, the data comprised 214 points (122—pre-law; 92—after-law), each corresponding to one day. No autocorrelation was observed. We used a bootstrap model with 1000 replications of the main model with randomly drawn samples with alpha = 0.05. We used “its.analysis” package in R [52] for this analysis.

## Results

### Dynamics of the network development

**Fig 2:**
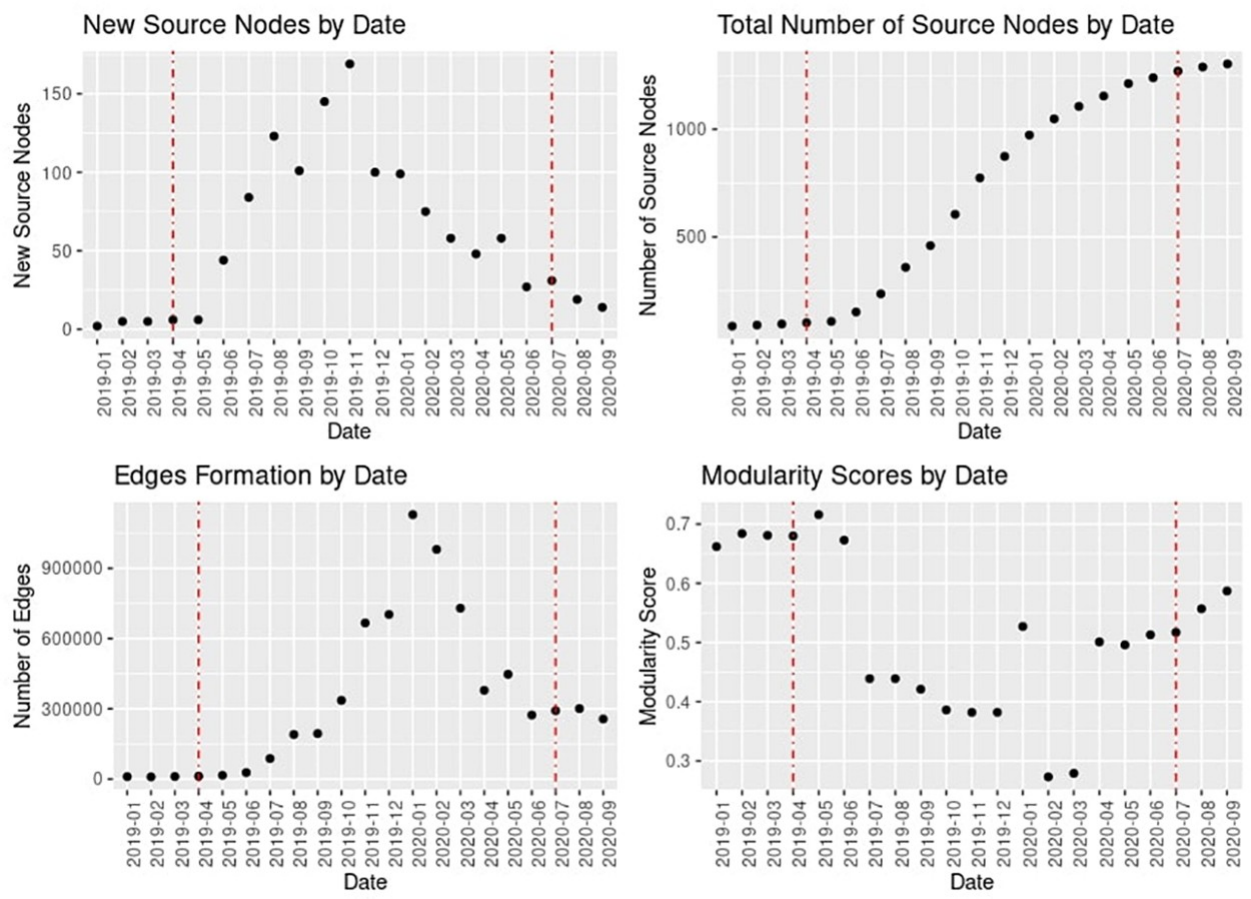
Dynamics of network development. Red lines correspond to: the announcement of the Anti-Extradition Bill in the end of March 2019, and the enactment of the National Security law in July 2020.

The rapid growth of the network started in June 2019, as reflected by the spike in the number of new Telegram channels (Fig 2). The growth rate in terms of the number of new channels peaked in November 2019, the most heated period of the protests in Hong Kong. Since then, the growth rate has decreased, however new channels are still being added every month, though in lower numbers than in summer and fall of 2019 (Fig 2).

The number of edges in the network (Fig 2) follows a slightly different pattern. The amount of connections in the form of mentions or reposts between Telegram channels started surging in July 2019, thus slightly lagging behind the spike in the number of new channels. It peaked around January 2020—two months after the peak in the number of new channels. Overall, the pattern of edge formation closely resembles that of new channel formation with the spikes in the latter preceding those in the former by 1-2 months.

#### Network fragmentation

We have computed modularity scores for each month between January 2019 and September 2020 to assess the level of network fragmentation in each month. The distribution of the scores is depicted in Fig 2. In the beginning of 2019, the network was highly fragmented with the modularity scores for each month from January 2019 to June 2019 being above 0.6. In practice, modularity score values above 0.5 are a significant departure from what could be observed in a random network, thus indicating the presence of a strong community structure, and, consequently, of structural holes [24, 53]. In July 2019, the modularity score fell to below 0.5, suggesting that the network became more cohesive between June and July 2019.

Our observation about the drastic decrease in the level of network fragmentation between June and July 2019 led us to check which channels have contributed to the increased coherence by connecting the most active political community to the others in the network. For this, we calculated brokerage scores for all the nodes (channels) in this most active community in June 2019. The most active political community was inferred from the network structure—i.e., we have qualitatively examined the biggest (by the number of nodes) communities in the network, and qualitatively checked the nodes with the highest authority and hub scores in each of them. The biggest community by the share of nodes (comprising 20.21% of all nodes in the June 2019 network) was identified as highly politically active, with some of its key nodes turning into major actors—by hub or authority scores—in the overall network at later stages. For each node in this community, we calculated brokerage scores corresponding to one of the following roles [54]:

- Gatekeeper role—brokers who serve as bridges between their own community and the others through incoming contact.
- Representative role—bridges between their own community and the others through outgoing contact.
- Liaison role—bridges between nodes from two groups they do not belong to.
- Coordinator role—facilitate contact between two nodes in their own group.

The first three roles described can be referred to as global brokers as they facilitate connections on the overall network level. The last role refers to local brokerage—coordinators facilitate connections between nodes in their own community.

**Fig 3:**
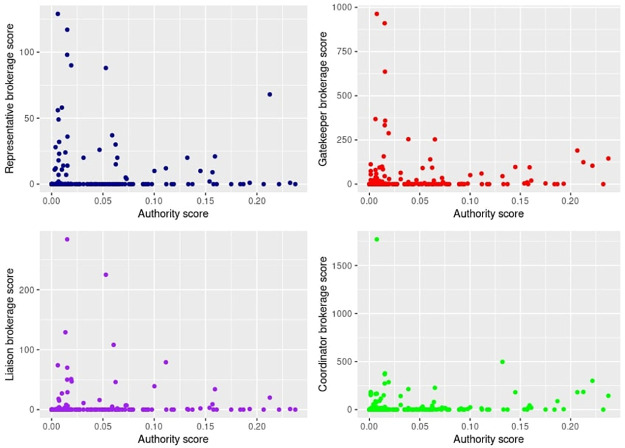
Association between authority scores and brokerage scores for the most politically-salient community in June 2019.

We then calculated the association between the brokerage scores and authority and hub scores. We find that there is no association between global brokerage and authority or hub scores. That is, channels that helped mediate contact between the political community in June 2019, at the start of the political organization on Telegram, were not important in terms of citation network (see Fig 3 for the illustration of association between authority scores and brokerage scores). However, there is a weak association between local brokerage scores and authority meaning that highly authoritative channels in the network were important for establishing contact between other channels in the politically active community. Still, the absence of association between global brokerage and hub/authority scores suggests that in June 2019, at the beginning of the network evolution, the connection of the political community to the others was facilitated predominantly by less prominent channels.

### Content analysis

**Fig 4:**
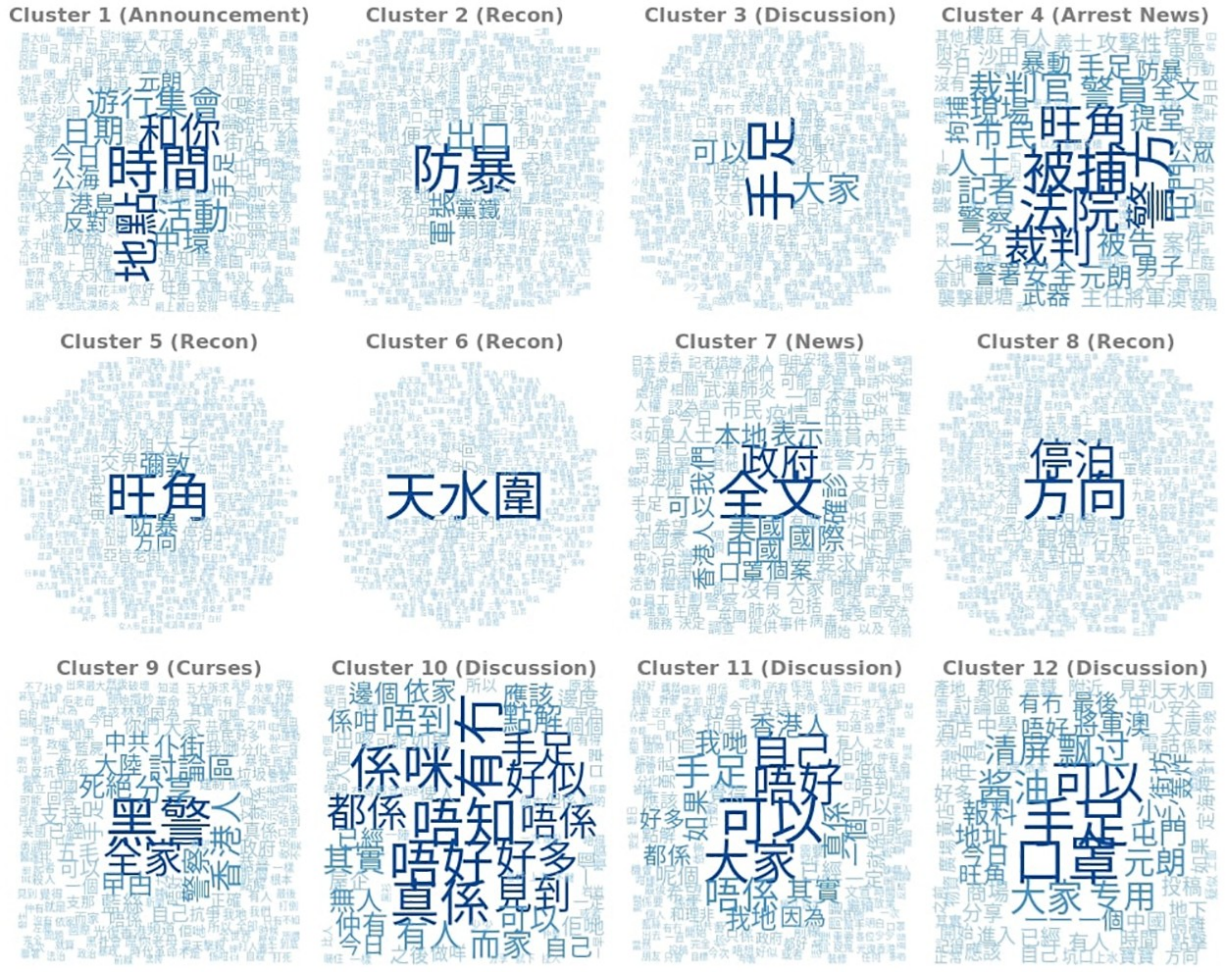
Wordclouds corresponding to 12 topic clusters.

**Fig 5:**
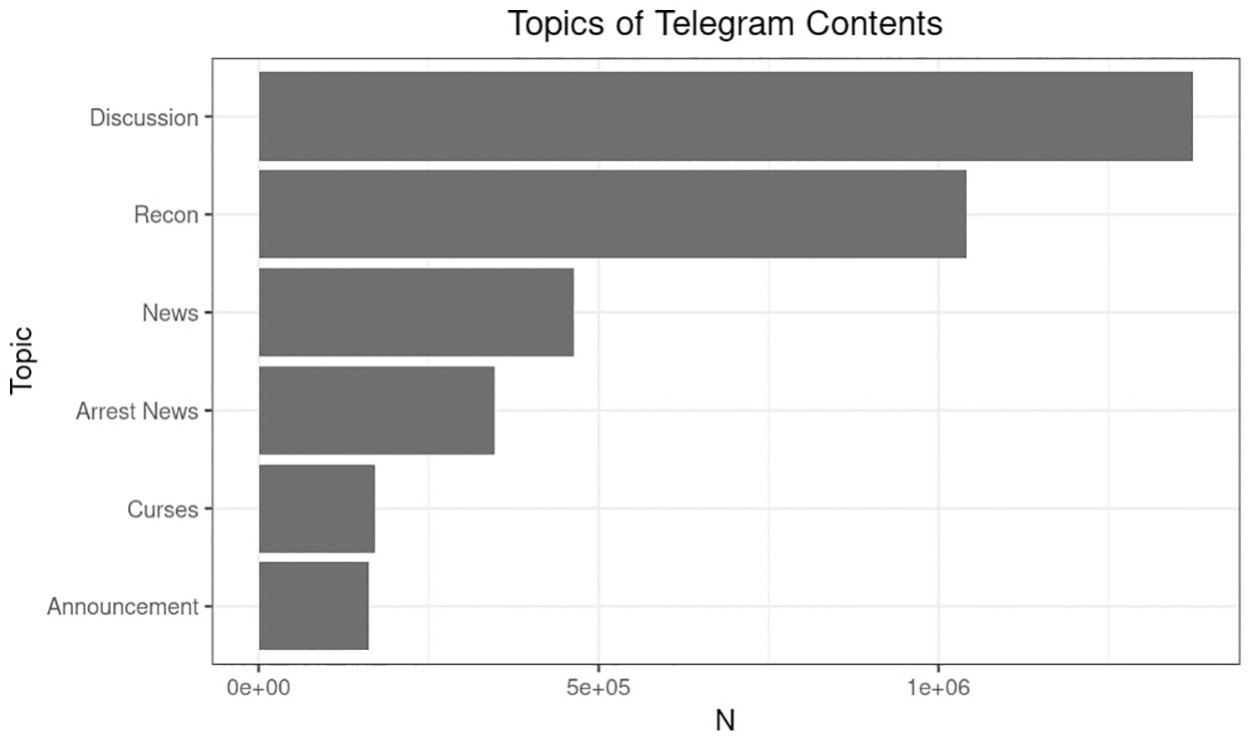
Prevalence of topics in the analyzed Telegram content.

In Fig 4 the top words of each topical cluster are displayed with English translations of the top 10 words listed in Table 1. We classified the clusters into six topics: Announcement, Recon, Discussion, News, Arrest News and Curses and also measured their prevalence across time and channel types. Announcement contains messages about upcoming protests and events. Recon reports information about the spotting of police officers and vehicles. The messages usually contain precise information of the location, time, and amount of police force (For example, “Tin Shui Wai 1706 Crossroad between XX and XX. 10 riot police has been deployed”). Discussion contains deliberations of protest tactics and also discussions about current affairs. Arrest News contains news on recent arrests, outcomes of trials, and court judgements. News contains links and extracts from news reports and articles as well as the discussion around them. The topic of Curses contains curses towards the “black cops”, a term generally used to refer to the police officers who have committed police brutality and violated human rights during the suppression of protests. Common slurs such as “death to the whole family of the black cops” (黑警死全家) and “Pokkai” (仆街), a common Cantonese insult phrase that can be translated as “bastards”. The prevalence of topics is shown in Fig 5.

**Table 1:** Top words per topic cluster.

Cluster	Translation of Top Words
Announcement	
1	Time, Location, With You, Activity, Assembly, Procession, Date, Central, Today, High Seas
Recon	
2	Riot, Exit, Military uniform, Party iron, Plainclothes, Tseung Kwan O, Causeway Bay, Landing, Central, Direction
5	Mong Kok, Nathan Road, Riot Police, Direction, Prince Edward, Junction, Yau Ma Tei, Tsim Sha Tsui, Parking, Argyle Street
6	Tin Shui Wai, direction, Yuen Long, Tuen Mun, Mooring, Doghouse, Light Rail, To, Ginza, Tipping
8	Direction, Parking, Flashlights, Kwun Tong, Outside, Travel, Sham Shui Po, Wanchai, Causeway Bay, Uniform Police
Discussion	
3	Brothers, Everyone, Can, Don’t, Help, Everybody, In Case, Under Arrest, Propaganda, Hope
10	Don’t Know, Have or Not, Don’t, Is It, In Fact, Many, Likely, Still, No, See
11	Can, Everyone, Don’t, Own, Brothers, No, Really, In Fact, We, Hong Kong People
12	Brothers, Masks, Can, Everyone, Clear, Yuen Long, Tuen Mun, Tipping, Address, Tseung Kwan O
Arrest News	
4	Under Arrest, Court, Police, Mong Kok, Judgement, Police Officer, Judge, Citizen, Person, Reporter
News	
7	Full Text, Government, United States, China, International, Show, Local, Diagnose, We, Can
Curses	
9	Black Cops, Whole Family, Hong Kong, Share, Forum, Policemen, Die, Cockroaches, Pokkai, Mainland

**Fig 6:**
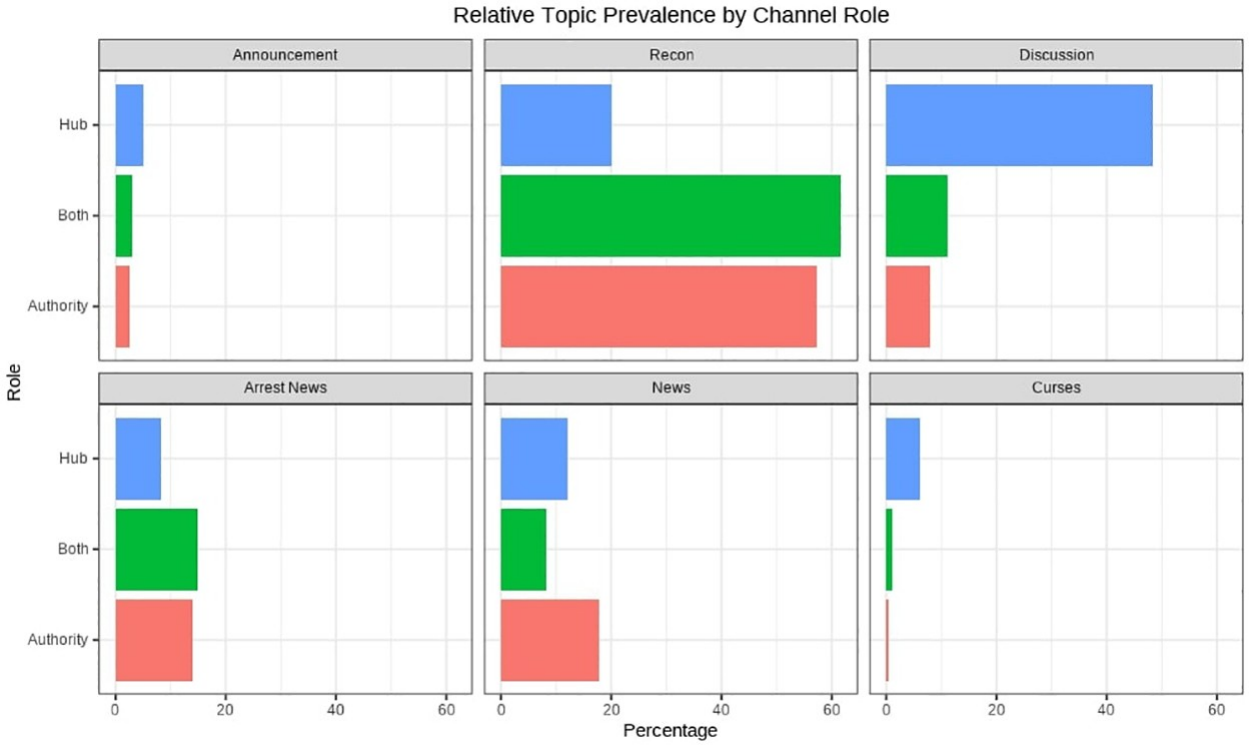
Relative topic prevalence by channel role.

Fig 6 shows the relative prevalence of topics among channels of different roles. Channels performing different structural roles within the Telegram citation network tend to address different topics. More than 50% of the messages posted by high-authority channels belong to the Recon topic while it is only around 20% for the hub channels. Authority channels also post slightly more News and Arrest News than the hubs. On the other hand, more than 40% of messages of hub channels belong to the Discussion topic. There are significantly more Curses messages among the hubs than among authorities although the topic only accounts for less than 10% of all messages. The results of the analysis suggest that Authority channels serve as the outlet for the flow of key information relevant to the protest while the Hub channels act as the platform for the deliberation among protesters.

**Fig 7:**
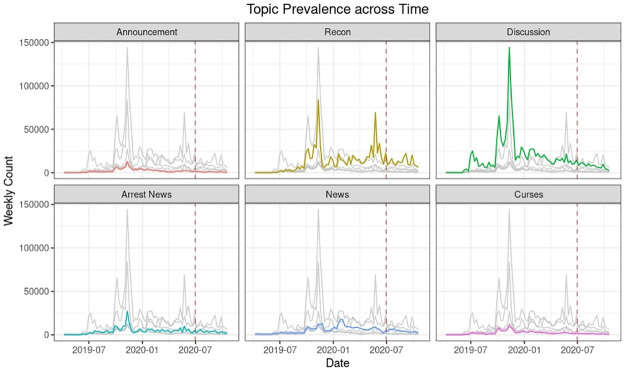
Topic prevalence across time. Red line corresponds to the introduction of the National Security Law.

The relative prevalence of different topics across time is demonstrated in Fig 7. The red dashed line denotes the date when the National Security Law was enacted. We observe two peaks of the Recon topic: one in mid-November 2019 during the city-wide strike and another in mid-May 2020 during the “Sing With You” protests after the ease in coronavirus lockdown. For the topic of Discussion, we can observe several overlapping peaks in the second half of 2019, leading to the climax in mid-November. All of Announcement, Arrest News, and Curses follow a similar trend with a peak around late 2019. The topic of News peaked in late January during the outbreak of COVID-19.

### Leaderless or not?

**Fig 8:**
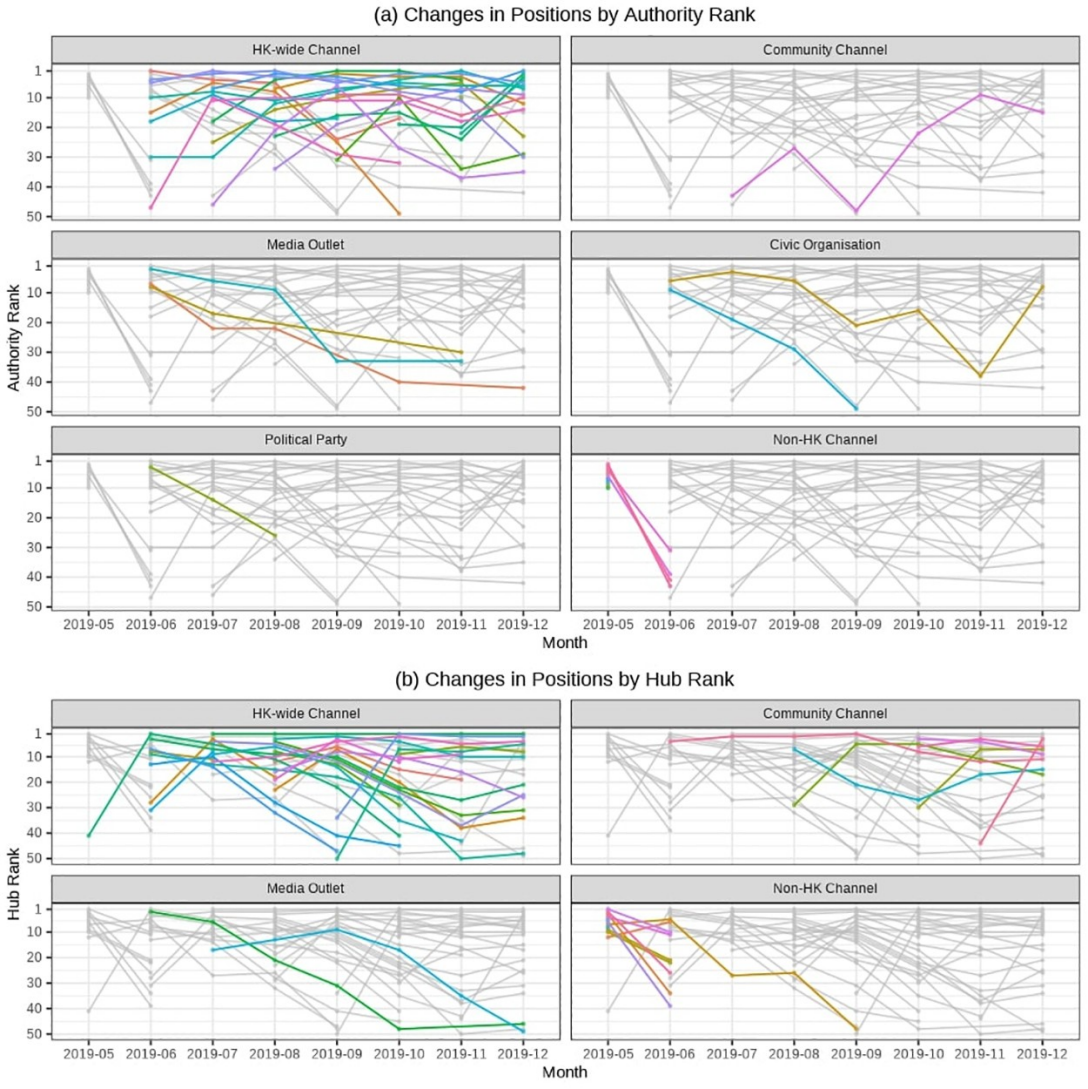
Changes in the channel/group positions by authority and hub score by month. To make the figure easier to interpret for those not familiar with individual Telegram channels and groups as well as to protect the identities of the most influential sources in light of the National Security Law, we report source types instead of particular source names. The classification of the types was done manually by one of the authors who is fluent in Cantonese and Mandarin and is highly familiar with the local context due to the focus of their research.

The changes in the positions of channels by authority score and hub score over time are presented in Fig 8. In these figures, we include only the data on top-50 positions for each month (i.e., if a channel dropped below top 50 in a given month, its data point for this month is omitted from the figures) to make the figures more illustrative of the dynamics around the top positions. Each line corresponds to one Telegram source (channel or public group).

As evident from Fig 8, there was high turnover in the positions of top sources by authority and hub scores. We suggest that this indicates the absence of specific leaders of the protest, at least in the form of organized groups or channels on Telegram. That being said, as we noted in the methodology section, the nature of the data does not allow testing for the presence of individual leader users who might have been behind different channels/groups who were switching in terms of the importance within the observed network. However, another observation that indirectly supports the idea about the decentralization of the protest and the absence of central leadership is that local community channels—i.e., those related to specific districts of Hong Kong,—were important as hubs and thus played a crucial role in disseminating protest-related information. Notably, in terms of both, hub and authority, Hong Kong-wide channels rather than media outlets, political parties or civic organizations (i.e. traditional leaders of past protests in Hong Kong including the Umbrella Movement) were predominantly important. However, the relative prominence of specific Hong Kong-wide sources was changing, highlighting the absence of long-term sustained leader channels/groups in the context of protest coordination and mobilization on Telegram.

The leaderless tendency is also reflected in the content of the messages, as the discourse suggesting a leaderless ideal was repeatedly iterated across channels. In many messages, the channels explicitly emphasized that they were not attempting to build a “central stage”, but to serve as a platform for discussion and information exchange. One typical example is the footer note attached to multiple messages by an authority channel: “This channel is not a central stage, we will not suggest or instruct anyone to take any action, our fellows should decide what actions to take”. Other channels, both authority and hub, also published similar messages during the movement.

### Cooldown after National Security Law

**Fig 9:**
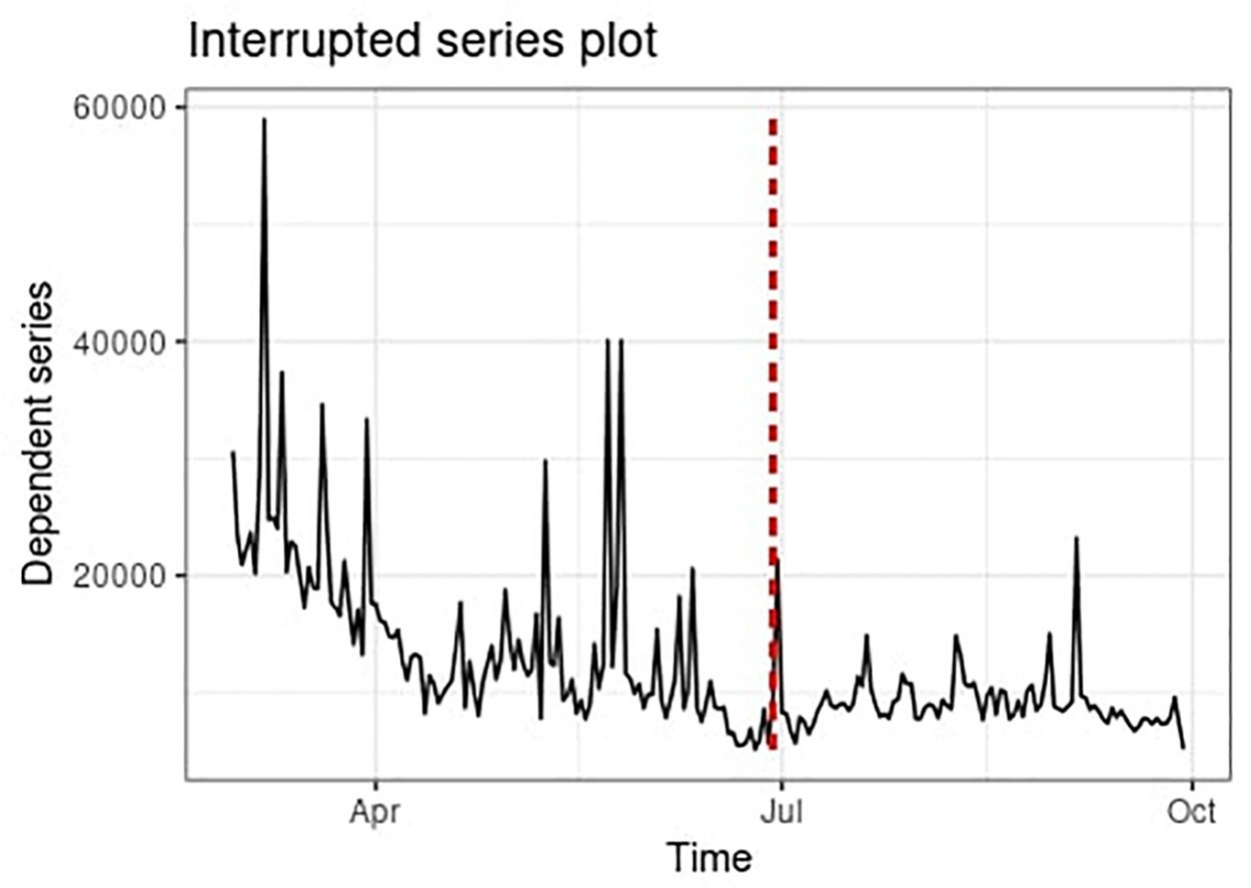
ITS plot of edge-creation activity on the Telegram network. Red line marks the introduction of the National Security Law.

We find significant (p<0.01: 95% CI, 3.3106-26.551) variation in the levels of activity between the 4-month period before the enactment of the National Security Law and the 3-month period after the introduction of the law. Before the introduction of the legislation, the mean daily rate of edge creation in the network was 14990.451(SD = 8271.572), and after 9229.065 (SD = 2605.587). The corresponding ITS plot is reported in Fig 9. Hence, the data suggests that the introduction of the National Security law has a triggered a significant decrease in the activity of the observed Telegram network. The selection of the time period for the analysis also allows us to account for the potential effects of the COVID-19 pandemic (i.e., that the activity had already decreased in the early months of 2020 as the protests could not be maintained due to the pandemic; still, we observe that the activity plunged further after the introduction of the law).

**Fig 10:**
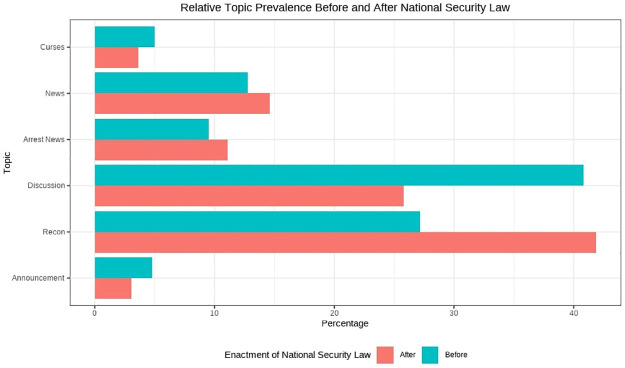
Topic prevalence before and after the introduction of the National Security Law.

We have also analyzed the prevalence of different topics in Telegram content before and after the enactment of the Security Law (Fig 10). Most topics follow similar distribution before and after the enactment of the law, with the exception of Discussion and Recon. The evidence suggests that the Telegram users engaged relatively less in discussion about current issues and protest strategy and more in reconnaissance after the introduction of the law. However, it is important to note that the absolute amount of both Discussion and Recon after the Security Law was smaller than the amount before the Security Law. Further analysis when more data points become available would be needed to substantiate the observation.

After the enactment of the National Security Law, we also observe discussion about the security measures in response to the risk of arrests, including tips and measures to protect privacy, guidelines on using prepaid sim card, Google Voice, and VPN services to avoid tracking, ways to change Telegram setting to make the account not identifiable by phone number, and how to completely wipe chat record from cloud storage and mobile phone. While some of the guidelines were drafted before the law, they were recirculated after the enactment.

## Conclusion and discussion

Our analysis showed that Telegram became popular with Hong Kong’s political activists in June 2019, when major protests against the Extradition Bill occurred with protesters surrounding the LegCo and blocking the introduction of the legislation. Since then the Hong Kong Telegramsphere was swiftly growing throughout 2019 with the growth peaking in the fall simultaneously with the most active protest period.

Importantly, the citation network on Telegram, fragmented in the beginning, rapidly became cohesive, thus fostering the efficiency of the spread of information. In the case of Hong Kong protests, the increased coherence of the network ensured the efficient diffusion of information among local and city-wide communities. Channels and groups that connected the political community to other communities in the early stage of the network formation might have fostered the increased cohesion. Notably, such broker channels were not particularly influential in the Telegramsphere in terms of their hub and authority scores (they were neither active information spreaders nor frequently cited by other important channels themselves). Still, their role in reaching out to other communities might have been crucial for the subsequent efficient spread of information in the network, suggesting that in the case of Telegram-based protests such “low key” actors can be of high importance. It would be worthwhile to assess the role of such brokers as well as the levels of network cohesion in other Telegram-aided protest movements within future research to establish whether our observations are context-specific or can be generalized to other movements on the platform.

Text analysis results demonstrate that Telegram was used by the protesters mostly to distribute information related to police presence and protest-related actions, as well as for deliberation. Channels and groups with high authority scores were dominated by information on protest-related events. In turn, those with high hub scores that aggregated such information from multiple high-authority sources, were used for discussion of further steps—possibly, based on the information about police presence and other events coming from high-authority sources. Thus, these two types of Telegram channels and groups worked in synergy. The type of information that was spread in Hong Kong Telegramsphere is precisely the information that scholars find important for protest mobilization [22]. Along with the fact that the observed network was cohesive making the spread of information efficient, this provides credible evidence that Telegram, as suggested by the media before [10], played a crucial instrumental role in the organization of 2019 protests in Hong Kong.

The analysis also reveals that the protests were de-facto leaderless, as previously claimed [21], with different channels switching in terms of the dominance in the network every month. We would like to reiterate, however, that the nature of the data allows only testing for the presence of sustained leadership (in the context of coordination and mobilization) constituted by group chats and/or public channels. We, thus, can not track the influence of individual users who might have administered specific channels, neither can we follow discussions in private groups. This is a major limitation that, nonetheless, can not be overcome due to the specifics of Telegram functionality, and should be accounted for both, when interpreting the results, and when planning Telegram-focused future studies.

Remarkably, local community channels and groups were of significant importance for the coordination and distribution of information in the network. Anecdotal evidence suggests that local group chats and channels also play an important role in Telegram-based protest mobilization in Belarus—activists even compiled a map listing local community Telegram sources [55]. This is an indication that Telegram might be a platform that fosters synergetic development of connective and collective action, promoting community building on the local level and the connections between such local communities. Seemingly, these are such communities that play crucial role in protest coordination and deliberation, without specific leaders. Further analysis of Telegram-aided protests in different contexts is necessary to scrutinize these claims and establish whether our observations hold true in contexts other than Hong Kong.

Finally, interrupted time series analysis results show that the National Security Law introduced in July 2020 has triggered a significant decrease in Telegram-based activity. This is a troubling observation since it suggests that even an app that is somewhat immune to online censorship is of little help in the context of protest mobilization per se against a powerful autocratic state such as China, should it resort to harsh offline measures to curb protests. Nonetheless, such apps, if in fact inpenetrable by law enforcement, still can serve as important communication channels among pro-democracy-minded citizens in authoritarian states. Given that our analysis includes only public group chats and channels, it is impossible to say whether in this case the activity on Telegram really subsided in general or only shifted to private spaces that are more difficult to monitor by security services. Further, we have analyzed only a period of three months since the introduction of the Law. We believe that right now it is impossible to say how effective the Security Law is for curbing protest mobilization in Hong Kong in a long term perspective. A follow-up analysis of the developments will be necessary to assess this once more time passes since the introduction of the Law. Hong Kong has experienced harsh crackdowns before, for instance, after the Umbrella Movement of 2014, and nonetheless the protest movement in the city continued to evolve. It is possible that, following an initial cooldown and chilling effect, protest activities in Hong Kong—on Telegram or otherwise—will bounce back to pre-Security Law levels.
